# Supplementary material for: Direct retrieval of Zernike-based pupil functions using integrated diffractive deep neural networks
Source: Nat Commun. 2022 Dec 7;13:7531. doi: 10.1038/s41467-022-35349-4 (PMC9729581; doi:10.1038/s41467-022-35349-4)
Supplement: Supplementary file 1 — Supplementary Information [file 41467_2022_35349_MOESM1_ESM.pdf]

Supplementary Information

Direct retrieval of Zernike-based pupil functions using integrated diffractive deep neural networks

Elena Goi[1,2 *], Steffen Schoenhardt[1,2] and Min Gu[1,2 *]

Dr. Elena Goi, Steffen Schoenhardt, Prof. Min Gu

[1] Institute of Photonic Chips, University of Shanghai for Science and Technology, Shanghai 200093, China

[2] Centre for Artificial-Intelligence Nanophotonics, School of Optical-Electrical and Computer Engineering, University of Shanghai for Science and Technology, Shanghai 200093, China

E-mail: elenagoi@usst.edu.cn, gumin@usst.edu.cn

Supplementary Materials

Wave analysis

The ID_2N_2 can be considered a system composed by a series of layers, each consisting of $N \times N$ resolvable pixels that act as diffractive neurons able to receive, modulate and transmit a light field. Each diffractive neuron can be considered as a source of secondary waves with amplitude and phase determined by the product of the input field and the transmission coefficients of each diffractive neuron. The first layer under coherent illumination generates an input represented by a $N \times N$ complex-valued vector. In our case, the diffractive layers modulate the phase of the incoming image, and the detector placed at the output layer collects the result of the all-optical inference. The nonlinear activation function is implemented during the photoelectric conversion via the imaging sensor, when the output image containing the information on the original pupil phase is detected. The photoelectric non-linearity of the detector, is modelled as a ReLU function (Supplementary Figure 1). More in detail, the diffractive neurons of each layer are linked to the neurons of the neighboring layer through free-space wave propagation following the Rayleigh-Sommerfeld[1] diffraction equation in the far field regime, $w_i(x, y, z)$ where i represents the i-th pixel of a given layer of the system located at (x_i, y_i, z_i) position . The product of the input wave and the i-th pixel transmission coefficient (b_i) determine the amplitude and relative phase of this secondary wave. Based on this, at the output layer of the MLD, the output function $y_{out_i}(x, y, z)$ of the i-th pixel located at (x_i, y_i, z_i) position can be written as[2]:

$$y_{out_i}(x, y, z) = w_i(x, y, z) \cdot b_i(x_i, y_i, z_i) \cdot y_{in_i}(x_i, y_i, z_i), \qquad \text{(Eq. 1)}$$

where:

$$y_{in_i}(x_i, y_i, z_i) = \sum_k (w_k(x, y, z) \cdot y_{0_k}(x_i, y_i, z_i)), \qquad \text{(Eq. 2)}$$

$$b_i(x_i, y_i, z_i) = a_i(x_i, y_i, z_i)\; e^{\left(j\, \Phi_i\ (x_i, y_i, z_i)\right)}, \qquad \text{(Eq. 3)}$$

$y_{0_i}(x, y, z)$ is the complex field distribution at the input field, $a_{i_0}(x_i, y_i, z_i)$ is the amplitude coefficient of each diffractive neuron (in our case is constant and equal to 1), z = $\frac{\Phi}{2\pi} \cdot \frac{\lambda}{\Delta n}$, λ is

the wavelength, Δn is the difference between the refractive index of the ID_2N_2 material and air, and Φ is the phase value of each ID_2N_2 pixel.

The ID_2N_2 can work in reflection or in transmission mode, and both phase and amplitude values of each diffractive neuron can be adjusted, providing a complex wave modulation. In our work we consider only the case of coherent transmissive ID_2N_2 and phase only modulation. We consider the absorption of the photoresist negligible.

TensorFlow-based design and training

The implementation of the presented ID_2N_2s is a two-step process:

- first, we train the diffractive network in-silico, for which we assume the nonlinear activation function to be a ReLU function with optimized parameters (see Figure S1b for optimization).
- The computer-designed diffractive neural network is then printed to perform the experimental validation of the diffractive elements functionality. When optically characterising the functionality of the DNN, the ReLU function is approximated by the nonlinear photoelectric conversion of the CCD camera[3]. In this way, the presented ID_2N_2 directly produces an intensity map that represents the original pupil phase and therefore no post-detection computation is required.

For the in-silico training and numerical testing, we build the training and testing datasets generating random pupil phase functions using Zernike polynomials from Z_1 to Z_{14} (OSA/ANSI indices) (Supplementary Figure 2a) as they covered the strongest aberrations typically seen in microscopy[4].

The Zernike polynomials are defined as:

$$Z_n^m(\rho,\varphi) = R_n^m(\rho)\cos(m\varphi)\ , \qquad \text{(Eq. 4)}$$

$$Z_n^{-m}(\rho,\varphi) = R_n^m(\rho)\sin(m\varphi), \qquad \text{(Eq. 5)}$$

where m and n are nonnegative integers with $n \geq m \geq 0$ ($m = 0$ for even Zernike polynomials), φ is the azimuthal angle, ρ is the radial distance $0 \leq \rho \leq 1$, and $R_n^m(\rho)$ are:

$$R_n^m(\rho) = \sum_{k=0}^{\frac{n-m}{2}} \frac{(-1)^k (n-k)!}{k!\left(\frac{n+m}{2}-k\right)!\left(\frac{n-m}{2}-k\right)!}\, \rho^{n-2k} \quad . \qquad \text{(Eq. 6)}$$

The corresponding PSFs were generated by a fast Fourier transform (FFT) implementation of vectorial Debye theory[4,5]. The optical system chosen for our training consisted of infinity corrected objective lens with a numerical aperture (NA) of 0.25, a magnification of 10× operating in air and a point source of 100 µm diameter. The PSF was calculated at an axial offset from the geometric focus by a distance of +20 µm. For the optical system detailed above, a complete image of the PSF requires a lateral dimension of approximately 30 µm, that can be divided in 75x75 diffractive neurons with a diameter of 400 nm. The wavelength of the point source was 785 nm. The size of the diffractive layers has been chosen to match the size of the PSF generated by the 10×NA0.25 objective and the diffractive neuron size and number have been optimised for the chosen wavelength.

We obtained the ID_2N_2 design using TensorFlow (Google Inc.) framework. The forward propagation model and its corresponding TensorFlow implementation can be summarised by equations Eq. 1 – Eq. 3. The input field $y_{in}(x, y, z)$ carries the information related to the pupil phase of the PSF. Each diffractive neuron of the diffractive layer adds a phase delay to the transmitted signal to map each input key into a specific output pattern $y_{out}(x, y, z)$. The phase delay of each diffractive neuron is adjusted during the training. The calculated output intensity distribution and the target output intensity distribution were used to calculate the mean-squared-error loss function during the training. We used the stochastic gradient descent algorithm, Adam[6], to back-propagate the errors and update the ID_2N_2 to minimize the loss function. At the end of the numerical training, the phase delays of the diffractive neurons are optimised, and the design is fixed. The trained ID_2N_2 can be physically fabricated to perform the tasks for which it was trained for.

For the ID_2N_2 presented in this work, the size of the diffractive layers was chosen in order to match the size of the point spread function (PSF) generated by the 10× NA0.25 objective and the number of layers, the distance between layers, the diffractive neuron size and number have been optimised for the chosen operative wavelength of 785 nm. The choice of using four

diffractive layers is a compromise between the performance achieved by the diffractive networks and fabrication challenges. Adding more than four layers to the diffractive network does not improve much the performance of the network in terms of RMS error of the reconstructed phase pupil. At the same time, nanoprinting a diffractive network with more than four layers is quite challenging since a taller structure is more likely to be subject to distortion and printing errors.

The choice to use the ReLU function to approximate the nonlinearity of the detector is motivated by the response curve for a light sensitive detector module consisting of photoelectric conversion and electronic readout circuit, which can be divided into three parts: the dark area, the linear area and the saturation area, as shown by the figure below. The dark area of the response curve shows the detector modules response to very low light intensities; the output value of the detector module in this area is noisy and unpredictable. After the Noise Equivalent Exposure (NEE) point is reached, the output of the detector module becomes linear until a point called the Saturation Equivalent Exposure (SEE). At this point, increasing the light intensity results in a nonlinear increase in the detector module output. Our experiments happen before the SEE point and therefore the response function can be approximated by a ReLU function.

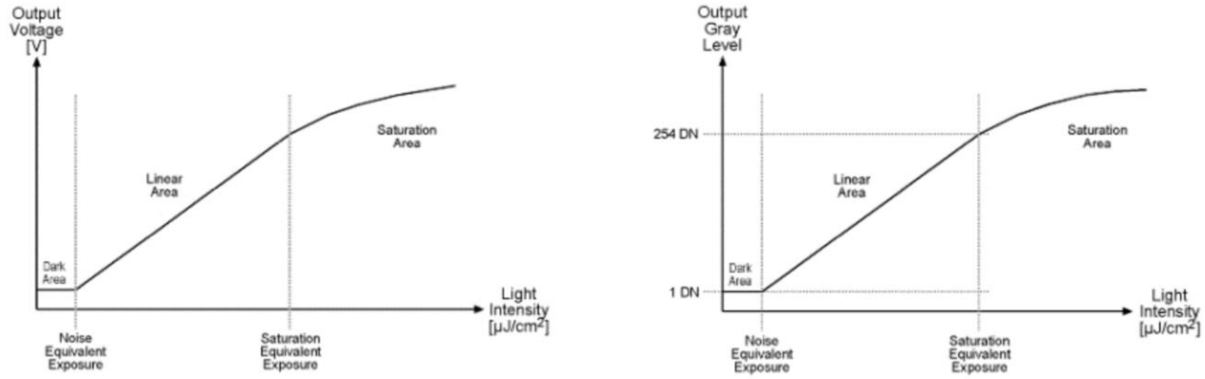

Typical response curves of a light sensitive detector module. Image adapted from [7].

During the training, we studied the relation between the brightness level of the output image and the system performance by tuning the parameter t of ReLU function (see Supplementary Figure 1). To adjust the brightness of an image, the value of all pixels can be changed by a constant. This can be achieved:

- In post processing, by adding a positive constant to all the image pixel values to make the image brighter or subtracting a positive constant from all of the pixel values to make the image darker. This can be achieved by applying a shifted ReLU function as transfer function.
- During the detection step, by adjusting the camera exposure parameters and in this way changing the NEE point.

Given the low impact of the parameter *t* on this specific task, for this qualitative experimental demonstration, we fixed exposure and gain conditions without optimizing the parameter t.

Vectorial two-photon nanolithography

The 3D-printed DN_2 were obtained by converting the calculated phase value of each pixel (Φ) into a relative height map $\Delta Z = \lambda\Phi / 2\pi\Delta n$, where Δn is the refractive index difference between the photoresist and air, and λ is the wavelength, in our case Δn=0.5 and λ =758 nm. The 3D model of the DN_2 was obtained using a Matlab code able to generate a point by point coordinate system.

The DN_2 were fabricated using a custom-made two-photon nanolithography (TPN)[8,9] system described in the Methods sections. TPN is a 3D nano-fabrication technique developed over the past two decades to what is now a well-established technology, with several commercially available systems on the market[10]. We printed the DN_2 using a dip-in approach[11,12] and a liquid commercial photoresist (IPS, Nanoscribe) compatible with fabrication on the packaged CMOS chip. A drop of IP-S was deposited on a glass coverslip or directly on CMOS sensor, and the photoresist was photopolymerised using the TPN method. The laser power used was 22 mW and the stage translation speed was 25 µ/s. After the nanolithography process, the samples were developed for 20 minutes in SU8 developer, rinsed with isopropanol and ethanol and then dried at room temperature. This procedure removes the unwritten material, leaving behind only regions where the focal spot has traced through and caused photopolymerisation to occur.

Usually, commercial TPN systems fabricate 3D objects by photopolymerizing consecutive layers of materials till the object is manufactured entirely. The minimum axial step (the

separation along the z-axis between two consecutive layers) available in commercial systems, like Nanoscribe, is 100 nm[10]. In this work, we used our custom made TPN system and employ a simplified vectorial printing approach to print the diffractive elements of the DN_2. We define the starting and the ending point for each rod that constitutes an artificial neuron, which is printed as a single rod, allowing for nanometric control of the length, enabled by the nanometric precision of the PI P-545.xC8S PInano Cap XY(Z) Piezo System. The lateral diameter of the artificial neurons was controlled by tuning the laser power and the printing speed.

Image post-processing

All the images used to characterise the ID_2N_2 are (sensor area of 2040 pixels × 2046 pixels, pixel size 5.5 × 5.5 μm², imaging wavelength region 400-900 nm). The output images of the DN_2 were magnified by a 4f system consisting of a 10× objective and a lens with a 150 mm focal length before being collected by the CCD camera.

CMOS prototype

Starting from a commercial Raspberry Pi Camera Module, as shown in Supplementary Figure 11a, we first mechanically remove the lens and lens housing using metal tweezers (Supplementary Figure 11a and Supplementary Figure 11b), before detaching the CMOS submodule (Supplementary Figure 11b top right), which carries a Sony IMX219 NoIR CMOS sensor, and cleaning the sensor surface with isopropanol. During this step one must pay close attention not to damage the wire bonds between the CMOS sensor and the PCB (printed circuit board) of the submodule. To print the ID_2N_2 the CMOS submodule was mounted on a microscope slide, commercial IP-S photoresist was deposited directly on the CMOS sensor, and photopolymerised using the TPN method in a dip in approach (as described in the *Vectorial two-photon nanolithography* section of this Supplementary Information). The fabricated ID_2N_2 as imaged through the CMOS sensor illuminated under an angle to the surface normal of the sensor is shown Supplementary Figure 11d, where one can clearly identify where the ID_2N_2 was printed on the CMOS sensor as square blocks highlighted in yellow color, while the shadow thrown on the sensor by the diffractive network under angled illumination is showing that it is indeed is a 3-Dimensional structure printed on the CMOS sensor. Supplementary Figure 11e shows a top-view of the printed diffractive network on the CMOS sensor.

To characterize the ID_2N_2 module, we re-attached the sensor submodule with the diffractive elements printed on the CMOS sensor to the camera base module and aligned the camera module in front of the 10X objective using a sample holder as shown in Supplementary Figure 11c. The results of the characterization of the ID_2N module prototype on the CMOS sensor for Z_0, Z_1, Z_2, Z_4 and $-Z_4$ Zernike polynomials are reported in Supplementary Figure 12, where the respective aberration functions are shown in Supplementary Figure 12a. Although the resolution is notably limited on the Sony IMX219 NoIR CMOS sensor, the PSFs recorded on this CMOS sensor shown in Supplementary Figure 12b are qualitatively in good agreement with the recorded PSFs as shown in Figure 4 and Figure 5 of the main text. The output of the ID_2N_2 module shown in Supplementary Figure 12c qualitatively allows for an estimation of the applied Zernike pupil phase function, including the sign of the defocus.

While the results presented in this section show that it is indeed feasible to fabricate a functioning prototype of our ID_2N_2 module integrated on a CMOS sensor, the challenges in the fabrication method as outlined above are manifold. For one, the mechanical removal of the lens and lens housing from the sensor module prior to fabrication is likely (~50%) to damage the sensor wire bonds, rendering the sensor useless. Although this would be not an issue in a more advanced fabrication scheme, where one starts off a 'bare' CMOS sensor, it makes the overall fabrication process described here unreliable. Further, with the TPN printing setup as described in the *Nanoprinting* section of the manuscript being configured for imaging of the substrate in transmission mode, identifying the surface of an opaque substrate is difficult, leading to potentially large errors (+/- 15 µm) in the distance between last diffractive element and detector plane, resulting in reduced performance of the ID_2N_2 module prototypes compared to the results of the benchtop demonstration shown in the main text.

Overall, this section shows the feasibility of fabrication of the ID_2N_2 prototype integrated on a CMOS sensor and its – while limited – functionality in directly determining pupil phase distortions, including the sign of the defocus aberration, from an incident point spread function.

Supplementary Figures

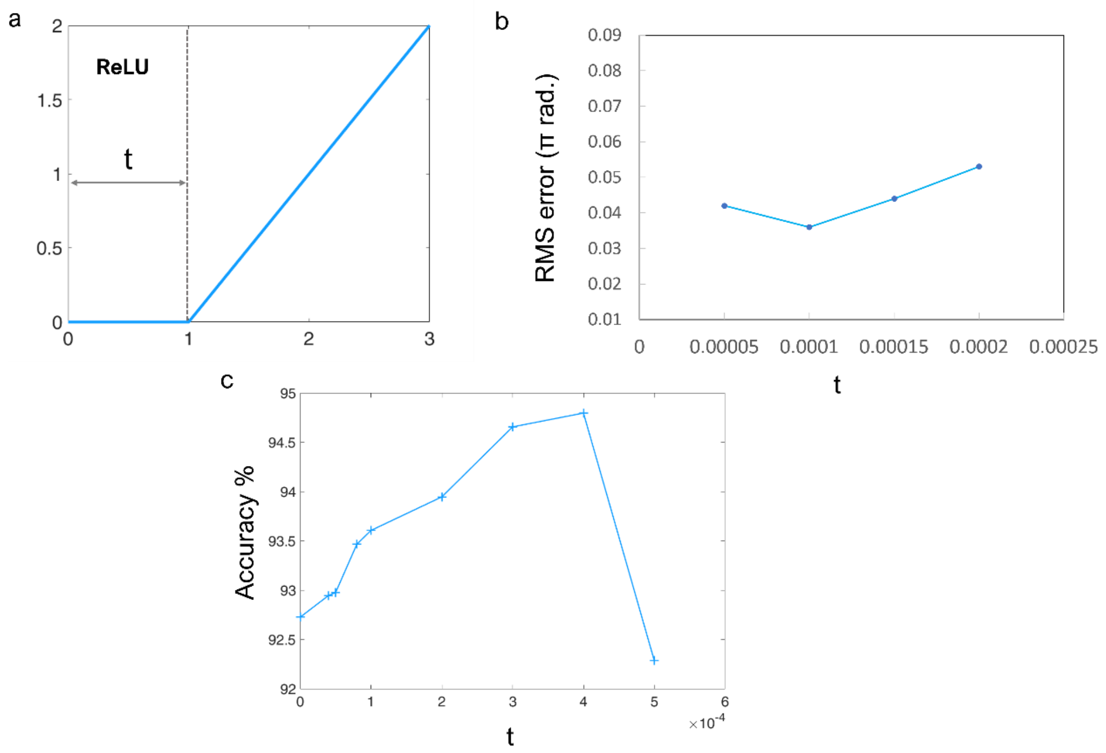

Supplementary Figure 1. **a**) Example of nonlinear ReLU function. The parameter *t* highlighted in the plot corresponds to the dark area of a light sensitive detector module response curve. **b**) Plot of the average RMS error as a function of the parameter *t*, for direct phase retrieval of single Zernike polynomial task performed by a four-layer diffractive neural network. **c**) Plot of the classification accuracy as a function of the parameter *t*, for handwritten digit classification task performed by a five-layer diffractive neural network[2]. When $t = 0$, the activation function is linear. When $t > 0$, the activation function is nonlinear. Each layer of the network has 6400 diffractive neurons, the ration between the wavelength and the pixel diameter is 1.875^2, the distance between layers is 40 wavelengths and the network was trained for 10 epochs with a learning rate of 0.001.

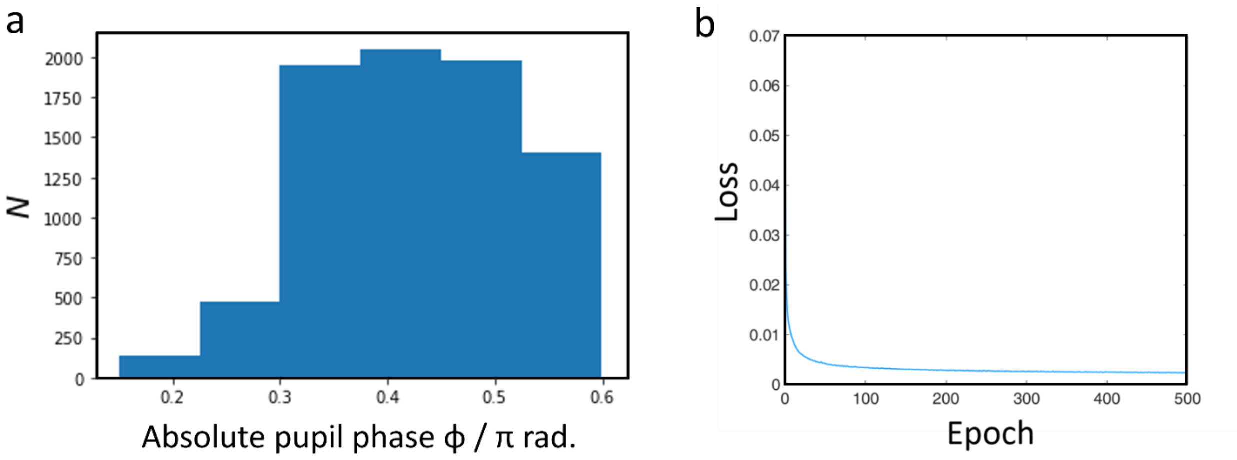

Supplementary Figure 2. **a**) Histogram of the occurrence of the absolute pupil phase between 0 and 0.6 pi in bins of 0.6 pi as extracted from the training dataset with 8000 pupil phase distributions. **b**) Training convergence plot for the ID^2N^2. The plot shows the loss values as a function of the epoch number.

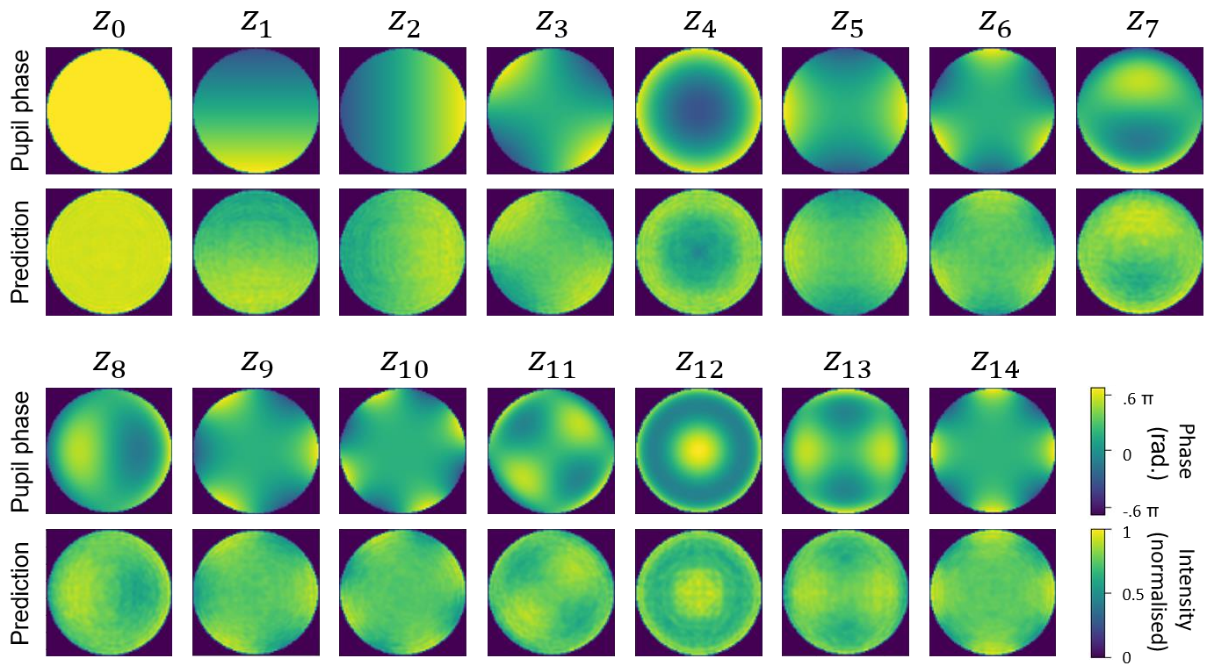

Supplementary Figure 3. Pupil phases and corresponding simulated ID_2N_2 outputs for the first 14 Zernike polynomials. The ID^2N^2 was trained for 500 epochs and optimised learning rate of 0.0001 with a training dataset of 8000 randomly selected polynomials between Z_1 and Z_{14} that were each assigned a random root-mean-square (RMS) magnitude between ± 0.6 π and the corresponding complex field PSF. The testing datasets comprise 500 PSFs per each Zernike polynomials. The operative wavelength λ was 785 nm, the distances from every layer of the ID_2N_2 was 31.4 μm and the absorption coefficient of the diffractive layer material α was approximated to 0. The PSFs were simulated at + 20 μm from the focal spot.

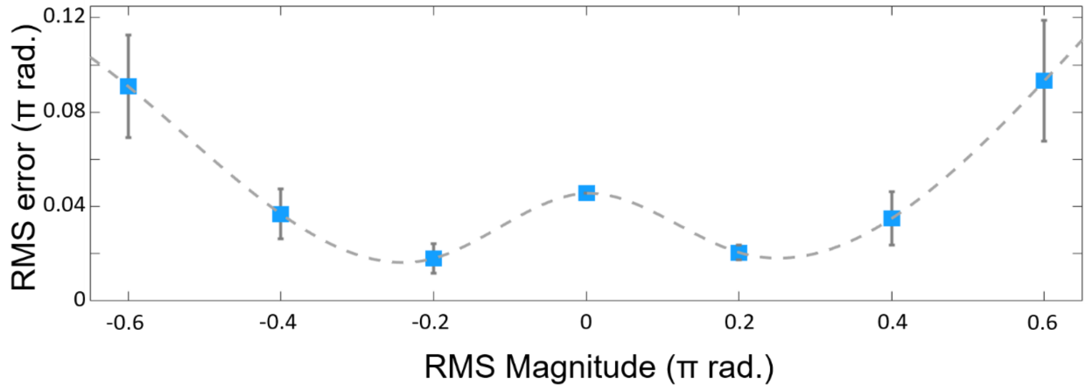

Supplementary Figure 4. Comparison of the RMS error between the pupil phase and ID_2N_2 outputs for sets of single Zernike polynomials (from Z_1 to Z_{14}) with RMS magnitude scanned from ± 0.6π. The RMS error for the single Zernike polynomials are reported in Supplementary Figure 5. The PSFs were simulated at + 20 µm from the focal spot.

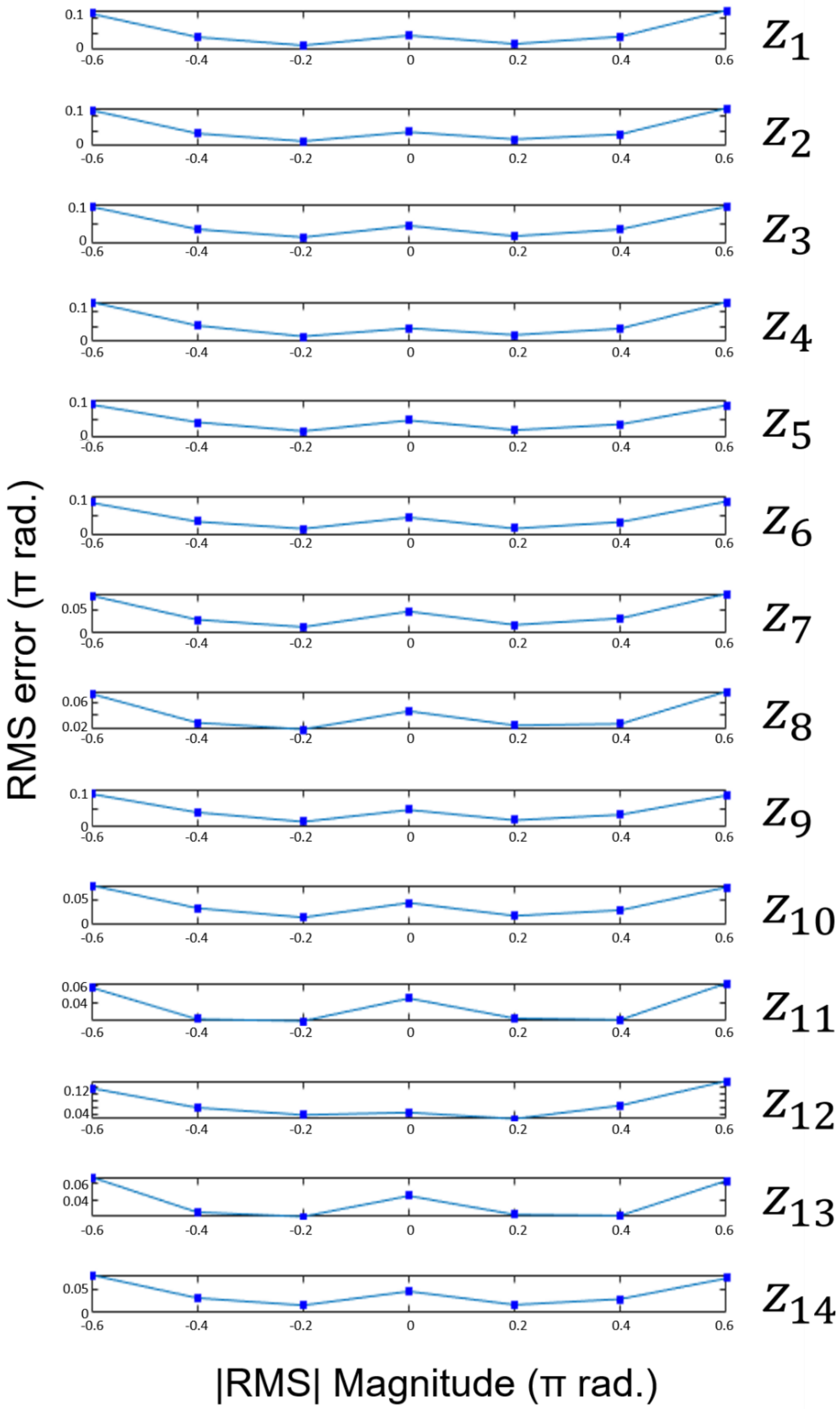

Supplementary Figure 5. RMS error (π radians) for single polynomial training.

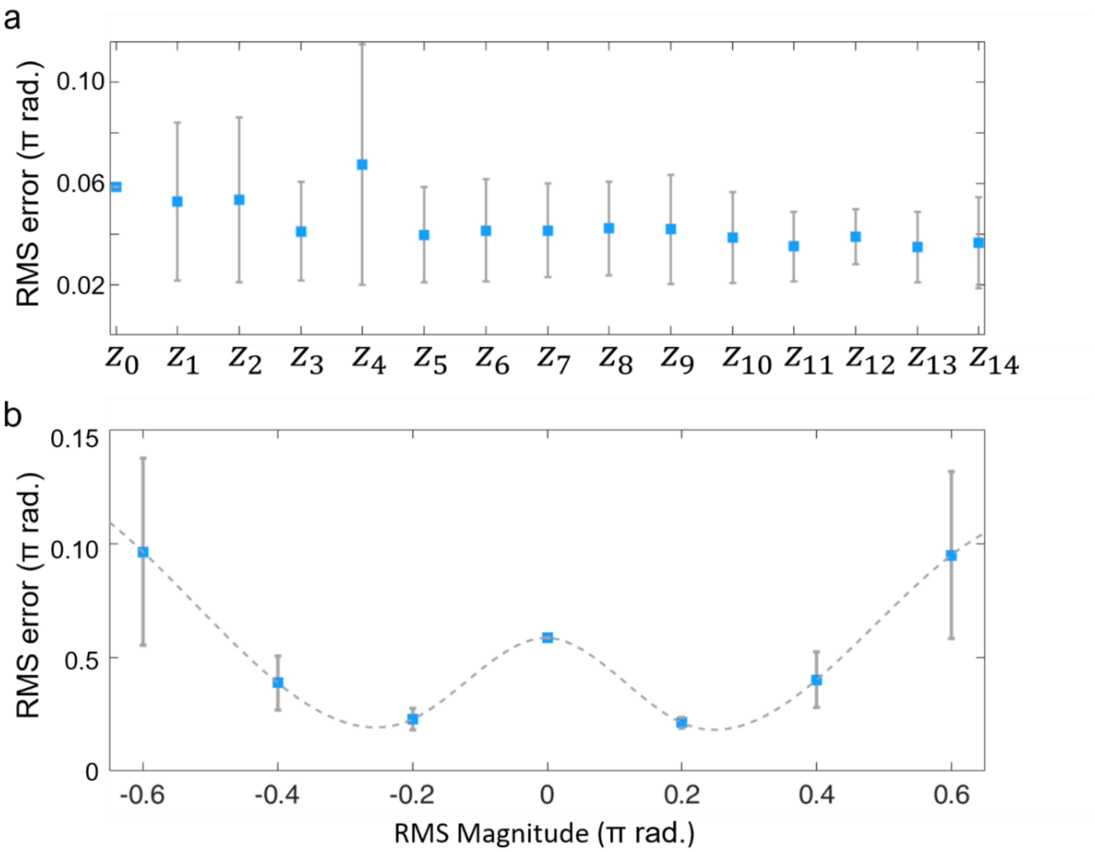

Supplementary Figure 6. Evaluation of the performance of ID_2N_2 trained using combination of Zernike polynomials. a) Comparison of the root mean square (RMS) error between the pupil phase and the ID_2N_2 outputs for the first 14 Zernike polynomial. b) Comparison of the RMS error between the pupil phase and the ID_2N_2 output for test data set of single Zernike polynomials (from Z_1 to Z_{14}) with RMS magnitude scanned from ± 0.6π. The RMS error for the single Zernike polynomials are reported in Supplementary Figure 5. The ID_2N_2 was trained for 500 epochs and optimised learning rate of 0.0001 with a training dataset of 8000 with randomized pupil phases comprised of combinations of Zernike polynomials (from Z_1 to Z_{14}) and the corresponding complex field PSF. Each of the 14 superimposed Zernike polynomials of the training and test datasets has a maximum amplitude that goes from -0.6 π radians to 0.6 π radians. They were added all up and the summation normalised again between ±0.6 π. In this way, the final maximum RMS magnitude does not exceed 0.6 π. The test data set is composed by 1000 randomized pupil phases comprised of combinations of Zernike polynomials (from Z_1 to Z_{14}). The operative wavelength λ was 785 nm, the distances from every layer of the DN_2 was 31.4µm and the absorption coefficient of the diffractive layer material α was approximated to 0. The PSFs were simulated at + 20 µm from the focal spot.

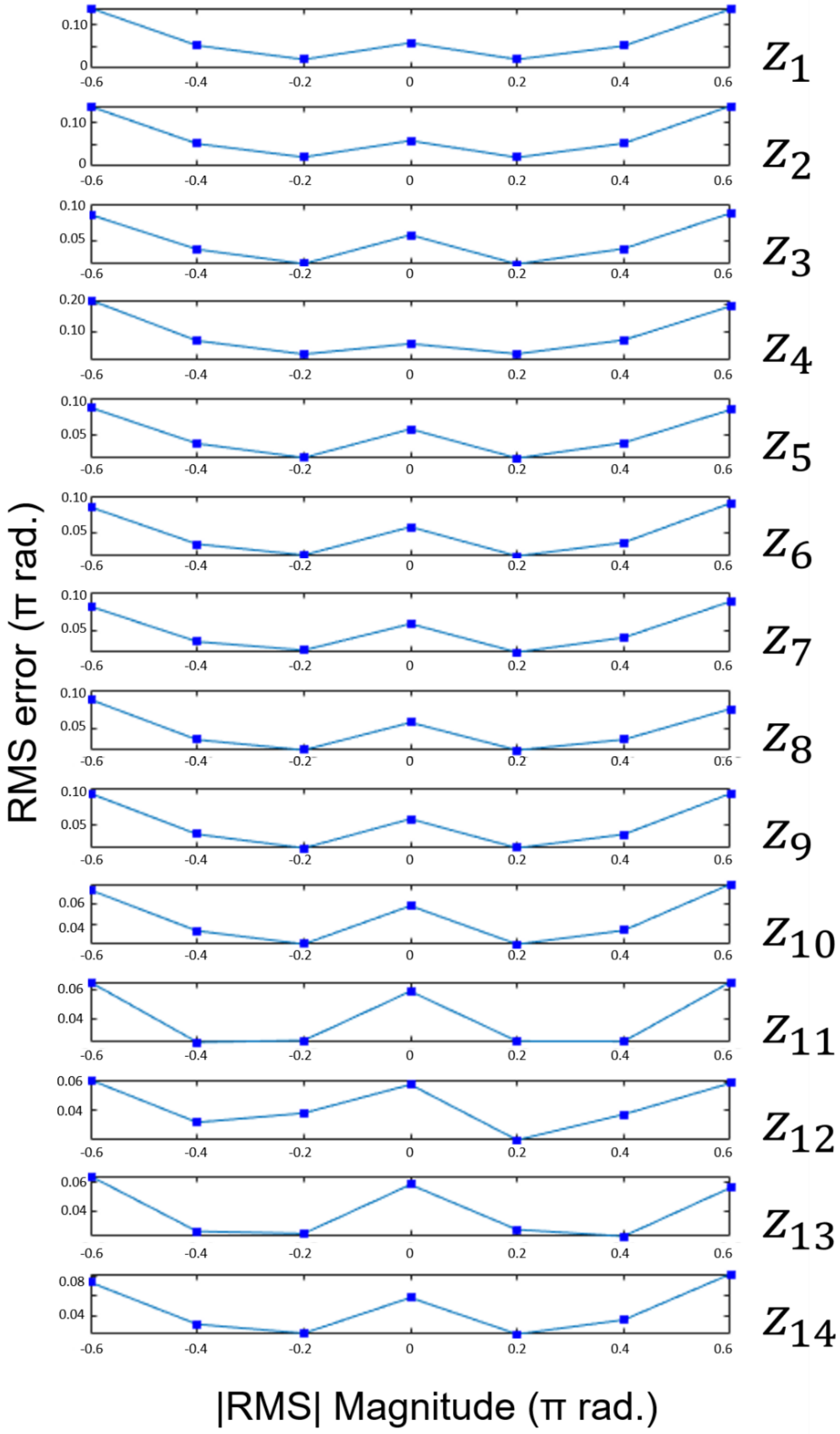

Supplementary Figure 7. RMS error (π) for combined polynomial training evaluated on single polynomials.

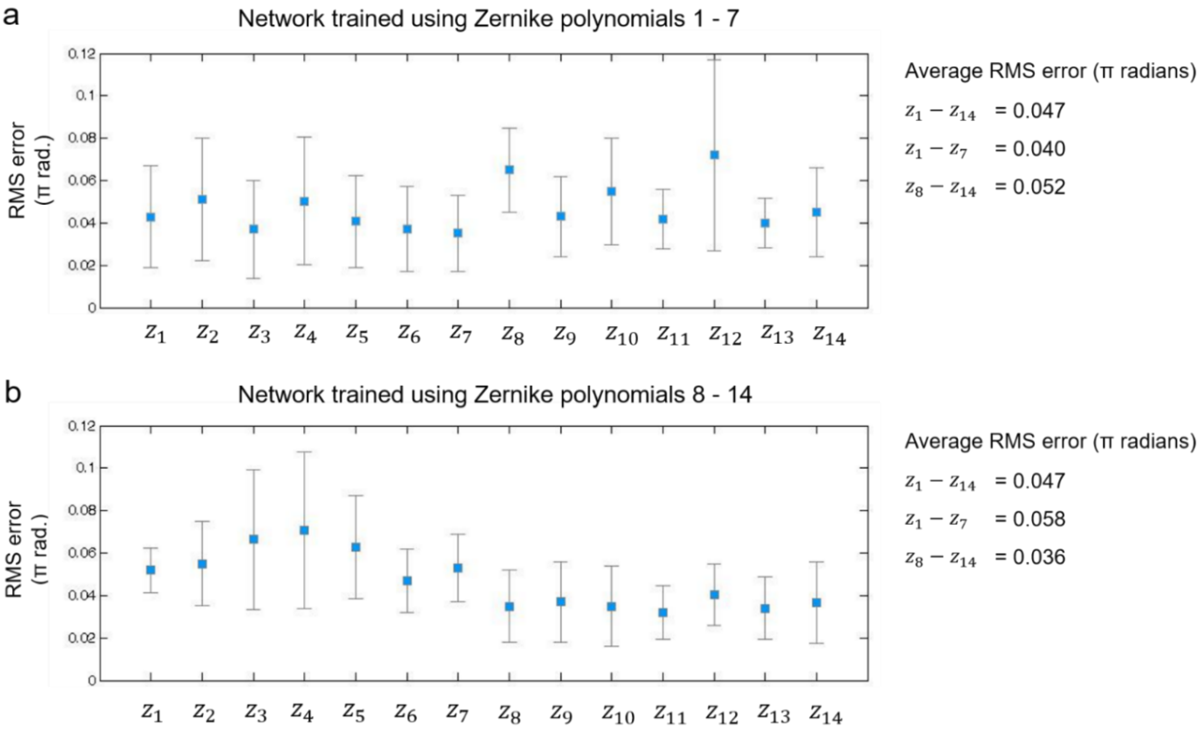

Supplementary Figure 8. Evaluation of the generalization ability of ID_2N_2. Comparison of the root mean square (RMS) error (π) between the pupil phase and the ID_2N_2 outputs for the first 14 Zernike polynomial for a network trained using a set of Zernike polynomial 1 to 7 (**a**) and a network trained with a set of Zernike polynomial 8 to 14 (**b**). The ID_2N_2s were trained in the same conditions of the network reported in Figure 3.

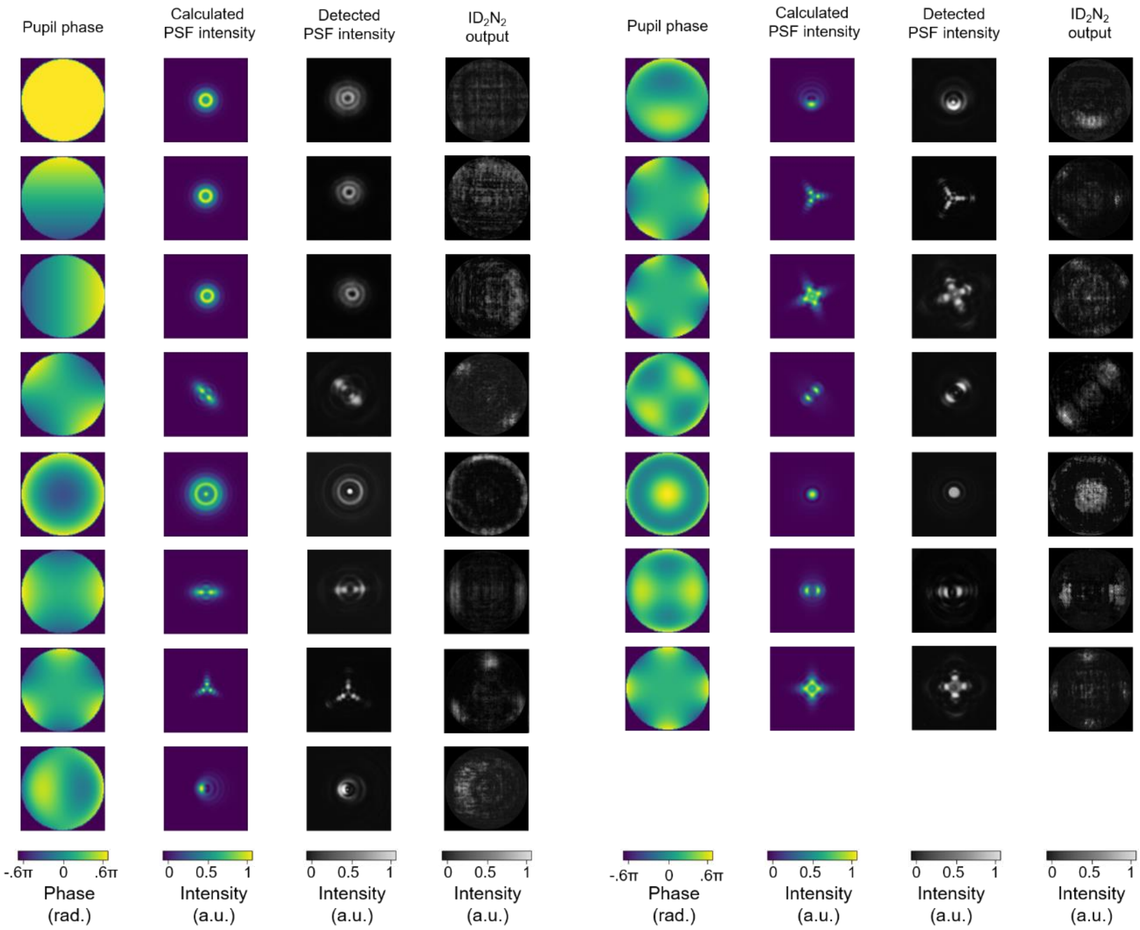

Supplementary Figure 9. Original pupil phases (with RMS absolute magnitude of 0.6 π radians) imposed to the point spread functions (PSFs), simulated PSF intensities, experimentally detected PSF intensities and experimentally detected ID_2N outputs for first 14 Zenike polynomials. The images of the experimentally detected PSF and wavefront predictions consist of 350×350 pixels (1.94 × 1.94 mm^2). Each DN_2 output image is plotted after the application of a 1.3 stretch to better highlight low intensity features. The PSFs were simulated and detected at + 20 μm from the focal spot of a 10X 0.25NA objective for a point source of 100 μm using a fast focus field method[5].

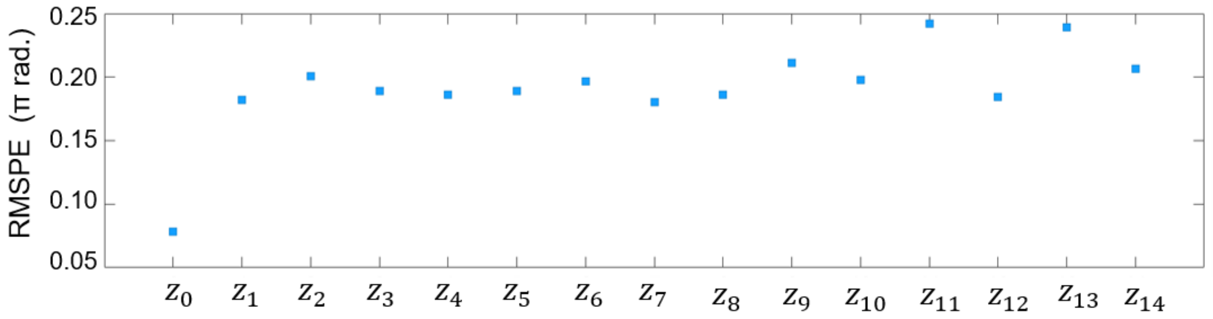

Supplementary Figure 10. Comparison of the root RMS error between the original pupil phase and the experimental ID_2N_2 outputs reported in Supplementary Figure 8 for the first 14 Zernike polynomials.

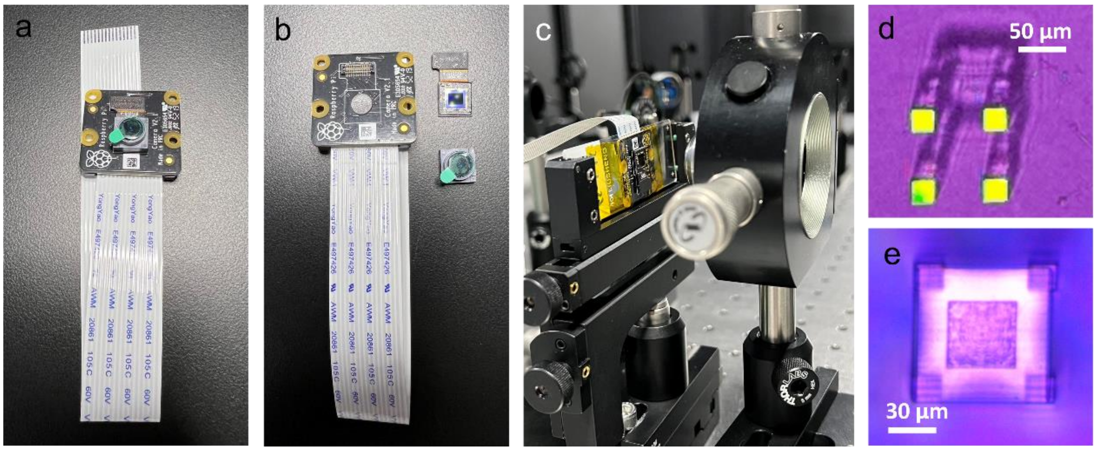

Supplementary Figure 11. CMOS prototype fabrication and characterization. **a**) Raspberry Pi Camera Module with Sony IMX219 NoIR CMOS image sensor. **b**) The image sensor is detached from the camera module and the lens and lens support are removed in preparation for the printing process. **c**) After the printing process, the image sensor is mounted on the camera module and positioned in front of the 10X objective. **d**) ID_2N_2 (bottom view) imaged through the Sony IMX219 NoIR CMOS image sensor illuminated under an angle to the surface normal of the sensor. **e**) Optical microscope image (reflection mode) of the ID_2N_2 (top view).

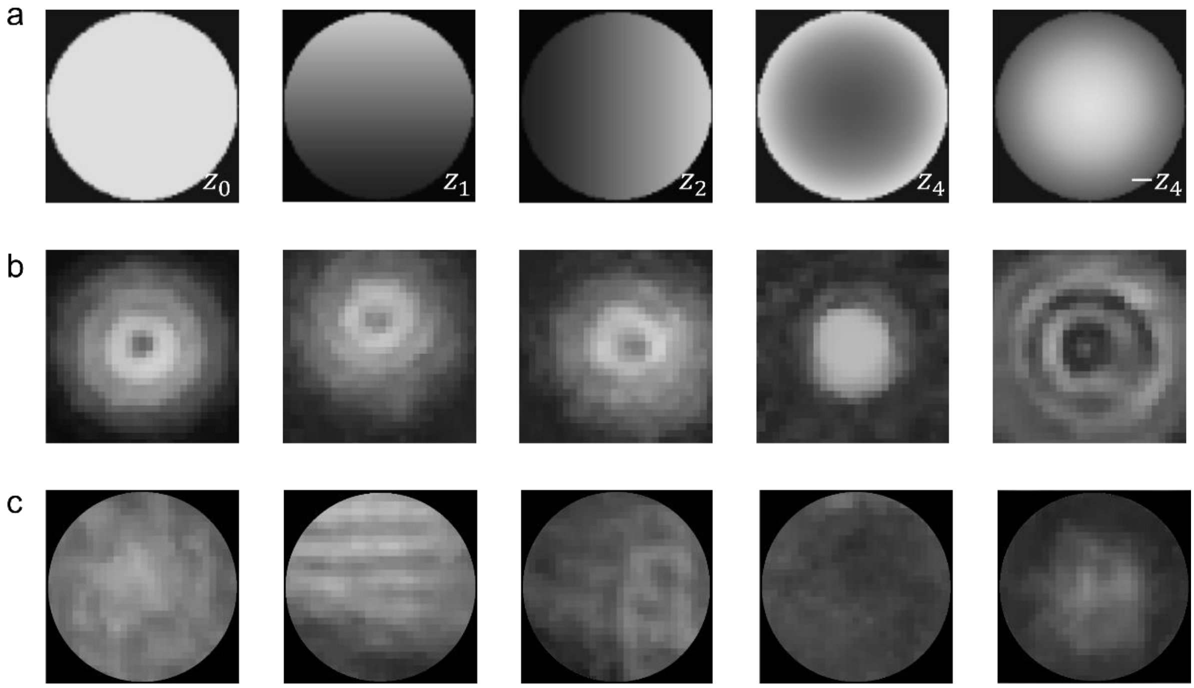

Supplementary Figure 12. CMOS prototype results. **a**) Original pupil phases (with RMS absolute magnitude of 0.6 π radians) imposed to the point spread functions (PSFs), (**b**) experimentally detected PSF intensities and (**c**) ID_2N_2 outputs for Z_0, Z_1, Z_2, Z_4 and $-Z_4$ Zernike polynomials experimentally detected by the Sony IMX219 NoIR CMOS image sensor. The ID_2N_2 was printed directly on the CMOS sensor and the images of the experimentally detected PSF and wavefront predictions consist of 27×27 pixels (30×30 μm^2).

References

1. Goodman, J. W. *Introduction to Fourier Optics. Third edition.* (Stanford University, 2005).

2. Lin, X. *et al.* All-optical machine learning using diffractive deep neural networks. *Science* **361**, 1004–1008 (2018).

3. Furber, S. Large-scale neuromorphic computing systems. *J Neural Eng* **13**, 051001 (2016).

4. Cumming, B. P. & Gu, M. Direct determination of aberration functions in microscopy by an artificial neural network. *Opt. Express* **28**, 14511–14521 (2020).
5. Leutenegger, M., Rao, R., Leitgeb, R. A. & Lasser, T. Fast focus field calculations. **14**, 4897–4903 (2006).
6. Kingma, D. P. & Ba, J. Adam: a method for stochastic optimization. arXiv:1412.6980 (2014).
7. What is sensitivity and why are sensitivity statements often misleading? https://www.baslerweb.com/en/sales-support/knowledge-base/frequently-asked-questions.
8. Turner, M. D. *et al.* Miniature chiral beamsplitter based on gyroid photonic crystals. *Nat Photonics* **7**, 801–807 (2013).
9. Goi, E., Cumming, B. & Gu, M. Impact of cubic symmetry on optical activity of dielectric 8-srs networks. *Applied Sciences* **8**, 2104 (2018).
10. Nanoscribe. https://www.nanoscribe.com/en/.
11. Bückmann, T. *et al.* Tailored 3D mechanical metamaterials made by dip-in direct-laser-writing optical lithography. *Advanced Materials* **24**, 2710–2714 (2012).
12. Goi, E. *et al.* Nanoprinted high-neuron-density optical linear perceptrons performing near-infrared inference on a CMOS chip. *Light Sci Appl* **10**, 40 (2021).
